# Pretreatment with an antibiotics cocktail enhances the protective effect of probiotics by regulating SCFA metabolism and Th1/Th2/Th17 cell immune responses

**DOI:** 10.1186/s12866-024-03251-2

**Published:** 2024-03-18

**Authors:** Jing Xu, Haoming Xu, Xue Guo, Hailan Zhao, Jiaqi Wang, Jianhong Li, Jie He, Hongli Huang, Chen Huang, Chong Zhao, Yingfei Li, Youlian Zhou, Yao Peng, Yuqiang Nie

**Affiliations:** 1 Department of Gastroenterology and Hepatology, the Second Affiliated Hospital, School of Medicine, South China University of Technology, Guangzhou, Guangdong China; 2 Department of Gastroenterology and Hepatology, Guangzhou First People’s Hospital, South China University of Technology, Guangzhou, Guangdong China

**Keywords:** Antibiotic cocktails, *Clostridium butyricum Miyairi588*, Gut microbiota, Short-chain fatty acids, Th1/Th2/Th17 response

## Abstract

**Background:**

Probiotics are a potentially effective therapy for inflammatory bowel disease (IBD); IBD is linked to impaired gut microbiota and intestinal immunity. However, the utilization of an antibiotic cocktail (Abx) prior to the probiotic intervention remains controversial. This study aims to identify the effect of Abx pretreatment from dextran sulfate sodium (DSS)-induced colitis and to evaluate whether Abx pretreatment has an enhanced effect on the protection of *Clostridium butyricum Miyairi588* (CBM) from colitis.

**Results:**

The inflammation, dysbiosis, and dysfunction of gut microbiota as well as T cell response were both enhanced by Abx pretreatment. Additionally, CBM significantly alleviated the DSS-induced colitis and impaired gut epithelial barrier, and Abx pretreatment could enhance these protective effects. Furthermore, CBM increased the benefit bacteria abundance and short-chain fatty acids (SCFAs) level with Abx pretreatment. CBM intervention after Abx pretreatment regulated the imbalance of cytokines and transcription factors, which corresponded to lower infiltration of Th1 and Th17 cells, and increased Th2 cells.

**Conclusions:**

Abx pretreatment reinforced the function of CBM in ameliorating inflammation and barrier damage by increasing beneficial taxa, eliminating pathogens, and inducing a protective Th2 cell response. This study reveals a link between Abx pretreatment, microbiota, and immune response changes in colitis, which provides a reference for the further application of Abx pretreatment before microbiota-based intervention.

**Supplementary Information:**

The online version contains supplementary material available at 10.1186/s12866-024-03251-2.

## Background

Inflammatory bowel disease (IBD) is a chronic and recurrent inflammatory disorder that includes ulcerative colitis (UC) and Crohn’s disease (CD) [1]. Currently, the commonly utilized drugs for IBD are ineffective and associated with adverse effects. Although environmental and genetic factors, intestinal epithelium, gut microbiota, and mucosal immunity have been variously implicated with IBD, its pathogenesis remains unclear [2]. With the development of sequencing technology, studies have reported a strong correlation between microbiota, immune response, and IBD [3, 4]. Specifically, the development of IBD is often accompanied by dysbiosis, and microbiological intervention strategies such as fecal microbiota transplantation (FMT) and probiotics can potentially reverse gut dysbiosis and regulate the dysfunction of an immune response [5].

It was observed that some certain bacteria were critical to maintaining intestinal homeostasis. *Clostridium butyricum Miyairi588* (CBM), a strict anaerobic Gram-positive strain that was first isolated in Japan, can relieve inflammation in mice with colitis by increasing protective cytokines [6]. CBM supplementation also alleviated barrier damage in a diarrhea model by enhancing lipid and amino acid metabolism [7], which has been clinically applied to patients with antibiotic-related diarrhea [8]. In addition, CBM also exhibits immunomodulation and anti-inflammation for gut diseases [9]. As a short-chain fatty acids (SCFA)-producing bacterium, CBM can degrade undigested fiber from the colon to produce benefit metabolites, thereby regulating the host’s gut homeostasis [10]. The butyric acid level characteristic of feces from patients with UC was decreased compared to that of the healthy control [11], which might indicate that complement CBM can elevate the level of SCFA to reduce the development of IBD. Thus, it was proposed that CBM is a potentially effective probiotic for IBD therapy. However, the exact mechanism by which CBM regulates the crosstalk of gut immunity and gut microbiota should still be clarified.

Although germ-free mice are a suitable experimental model for analyzing the effects of bacteria on host physiology and pathology, high maintenance costs and immune deficiency limit their application [12]. Antibiotic cocktail (Abx) pretreatment can simulate the intestinal state of sterile mice, and we previously observed that most gut bacteria can be removed by utilizing various combinations of antibiotics [13]. It was observed that Abx pretreated mice exerted a protective effect on hepatic injury and inflammation [14], which implied that gut microbiota depletion might exert a considerably higher therapeutic role on patients with UC. A recent study revealed that performing gut preparations prior to FMT, which includes taking amoxicillin, fosfomycin, and metronidazole for 2 weeks, can enhance symptoms in patients with UC [15, 16]. A meta-analysis indicated that Abx pretreatment before FMT can enhance the therapeutic effect on adult patients with UC [17]. However, whether pretreatment with Abx prior to the microbiota-based treatment leads to more optimal outcomes remains unclear and requires further analysis.

The aforementioned studies indicate the importance of clarifying the effect of Abx pretreatment on colitis and microbiota-based therapy. The current study was conducted to observe how Abx pretreatment prior to dextran sodium sulfate (DSS) -induced colitis can protect mice from colitis and to test the efficacy of CBM intervention on colitis with or without Abx pretreatment. Additionally, how the augmented protection by Abx pretreatment influenced the dysbiosis and dysfunction of gut microbiota and mucosal immune response were analyzed.

## Methods

### Establishment of a DSS-induced acute colitis animal model with or without ABx pretreatment

Thirty-six male SPF Balb/c mice (6–8 weeks old; weighing 24-26 g) were purchased from Guangdong Medical Experimental Animal Center. The animals were raised in a climate-controlled room at 25℃, and fed with ordinary chow. Six mice were housed per group in this experiment. The Ethics Committee approved animal experiments (Experimental Animal Certificate Number: SCXK [Guangdong] 2018-0002). The Abx included ampicillin (1 g/L), neomycin (1 g/L), metronidazole (1 g/L), and vancomycin (0.5 g/L). The antibiotics (all from Bomei, China) were weighed and added to the drinking water, which was autoclaved and replaced every two days for the following 4 weeks. Subsequently, the mice were given drinking water containing 3% DSS (MP Biomedical, America) for 7 days to induce acute colitis. Along with DSS intervention, live CBM (1 × 10^6^CFU/mL, 200 µl/mouse) was given orally once daily. The control group was given normal drinking water during the whole experiment and was gavaged with PBS to compare with the intervention groups. Moreover, the mice were anesthetized with an intraperitoneal injection of 90 mg/kg pentobarbital sodium at the endpoints [18]. The blood, colon, and caecal content samples of mice were collected when they under anesthesia. The ethics committee of the South China University of Technology approved the above animal experiments.

### Disease activity assessment

The mice were weighed daily during the experimental period, and the percentage of weight loss at the end of the experiment was scored as follows: 0 = 0 to < 1%, 1 = 1% to < 5%, 2 = 5% to < 10%, 3 = 10% to < 15%, and 4 = ≥ 15%. The diarrhea scores and bloody stool scores were calculated according to Berberat’s scoring standard [19]. The disease activity index (DAI) was calculated as one-third of the sum of weight loss scores, diarrhea scores, and bloody stool scores.

### Hematoxylin-eosin (H&E) staining

H&E staining was conducted as previously described [20]. Furthermore, the histological scores was blindly evaluated and recorded by two pathologists as reference [21]: (a) inflammation of lamina propria: 0 - infrequent, 1 - increased with some neutrophil infiltration, 2 - submucosal presence of inflammatory cell clusters, and 3 - transmural cell infiltration; (b) colon tissue damage: 0 - none, 1 - isolated focal epithelial damage, 2 - mucosal erosions and ulcerations, and 3 - extensive damage deep into the bowel wall [22].

### Bacterial preparation

CBM (Miyarisan Pharmaceutical Co. Ltd., Japan) was cultured with an MRS medium (Huankai, China) in an anaerobic environment (10% H_2_, 10% CO_2_ and 80% N_2_) for 14–16 h. The bacteria in the logarithmic growth phase were harvested and centrifuged at 3,500 rpm for 10 min at 4 °C, and the pellet was washed twice with cold sterile PBS and resuspended to a final concentration of 1 × 10^6^CFU/mL for subsequent experiments.

### Immunohistochemical staining

The colon sections were immuno-stained using primary antibodies for ZO-1 (ab216880, Abcam), OCCLUDIN (ab168986, Abcam), CLAUDIN1 (ab15098, Abcam), and MUC2 (ab272692, Abcam) as standard methods [23]. Two pathologists who were blinded to the treatment evaluated and scored the samples according to a previous standard [24].

### 16 S rRNA sequencing

Fecal DNA was isolated using the QIAGEN DNA stool Kit (Qiagen, Germany). The V3-V4 region of 16S rRNA was utilized using the primers 341F (5’-CCTACGGGNGGCWGCAG-3’) and 806R (5’-GGACTACHVGGGTATCTAAT-3’), and sequenced on the Illumina Novaseq 6000 platform (GENE DENOVO, Guangzhou, China). The GreenGene database was used to annotate the species [25]. Operational taxonomic units (OTUs) clustering was performed using UPARSE (Version 9.2.64) [26]. The α-diversity of the gut microbiome was measured using the Shannon and Chao1 indices. UniFrac distance was utilized to calculate the β-diversity, and visualized using the principal coordinate analysis (PCoA).

### Measurement of SCFA levels

The frozen stool samples were analyzed by gas chromatography-tandem mass spectrometry (Novogene, Beijing, China) to measure SCFA levels. Sample collection and pretreatment, standard preparation, peak detection, and data analysis were performed as per the protocols [27, 28].

### Quantitative real-time PCR

AG RNAex Pro reagent (AG21102, AG) was utilized to extract RNA, and it was reverse transcribed using a PrimeScript™ RT reagent kit with a gDNA Eraser (RR047A, Takara). QRT-PCR was performed using TB Green Premix Ex Taq II (RR820A, Takara) on qTOWER2.0 (Analytik Jena, Germany). Mouse ACTB was utilized as the internal reference gene. The 2^−ΔΔCt^ value was utilized to evaluate the relative expression of genes. See Table S1 for the primer sequences.

### Isolation of colon lamina propria cells

The colon tissue was gently minced and digested with collagenase IV (11,074,032,001, Roche) and DNaseI (10,104,159,001, Roche) at 37 °C for 20–40 min, 70 μm filtered, and further homogenized in a DPBS (without Ca/Mg) buffer (Gibco) supplemented with 1% bovine serum albumin (BSA; 11,021,037, Thermo Fisher Scientific). Moreover, the tissue was centrifuged and washed twice with DPBS to obtain the single-cell suspension.

### Flow cytometry analysis

Cell suspensions were counted and incubated in 1% BSA/DPBS for 30 min at 4 °C to block non-specific binding. The cells were incubated with FITC anti-mouse CD45 (157,214, Biolegend), APC-anti-mouse CD3 (100,236, Biolegend), PE/CY5.5 anti-mouse CD4 (100,434, Biolegend), and PE anti-mouse CD8a (100,708, Biolegend) and subsequently permeabilized with a suitable buffer (Biyuntian, China) ; moreover, they were incubated overnight with PE anti-mouse Foxp3 (126,404, Biolegend) and BV421 anti-mouse T-bet (644,815, Biolegend) antibodies at 4 °C for intracellular staining. To analyze cytokine secretion, the cells were stimulated with Monensin (420,701, Biolegend) and a cell activation cocktail (423,301, Biolegend), followed by surface staining, fixation, and permeabilization as described. The cells were the incubated overnight with PE anti-mouse INF-γ (505,808, Biolegend), BV421 anti-mouse IL-4 (504,119, Biolegend), and PE/CY7 anti-mouse IL-17 A (506,922, Biolegend) antibodies at 4 °C. The data was analyzed using Flowjo software (Tree Star Inc., San Carlos, CA).

### Statistical analysis

The statistical analysis was performed using SPSS software (version20.0, SPSS Inc., Chicago, USA), and the graphs were plotted using GraphPad Prism 8.0, R software (version 4.0.3) and Adobe Illustrator CC 2019. Measurement data are presented as the mean ± standard deviation, and counting data as the median (quartile distance). After testing for normal distribution and homogenous variance, two-tailed independent T-test and ANOVA were utilized to compare data of two or multiple groups respectively. Wilcoxon rank-sum test was utilized in inconsistent data. Based on the 16 S rRNA sequencing results, the diversity between the two groups was analyzed using the Wilcoxon rank sum test. Subsequently, the Kruskal–Wallis test was utilized to analyze PCoA results, where *P* < 0.05 denotes statistical efficacy.

## Results

### Abx pretreatment could protect the DSS-induced colitis mice from colon inflammation

**Fig. 1:**
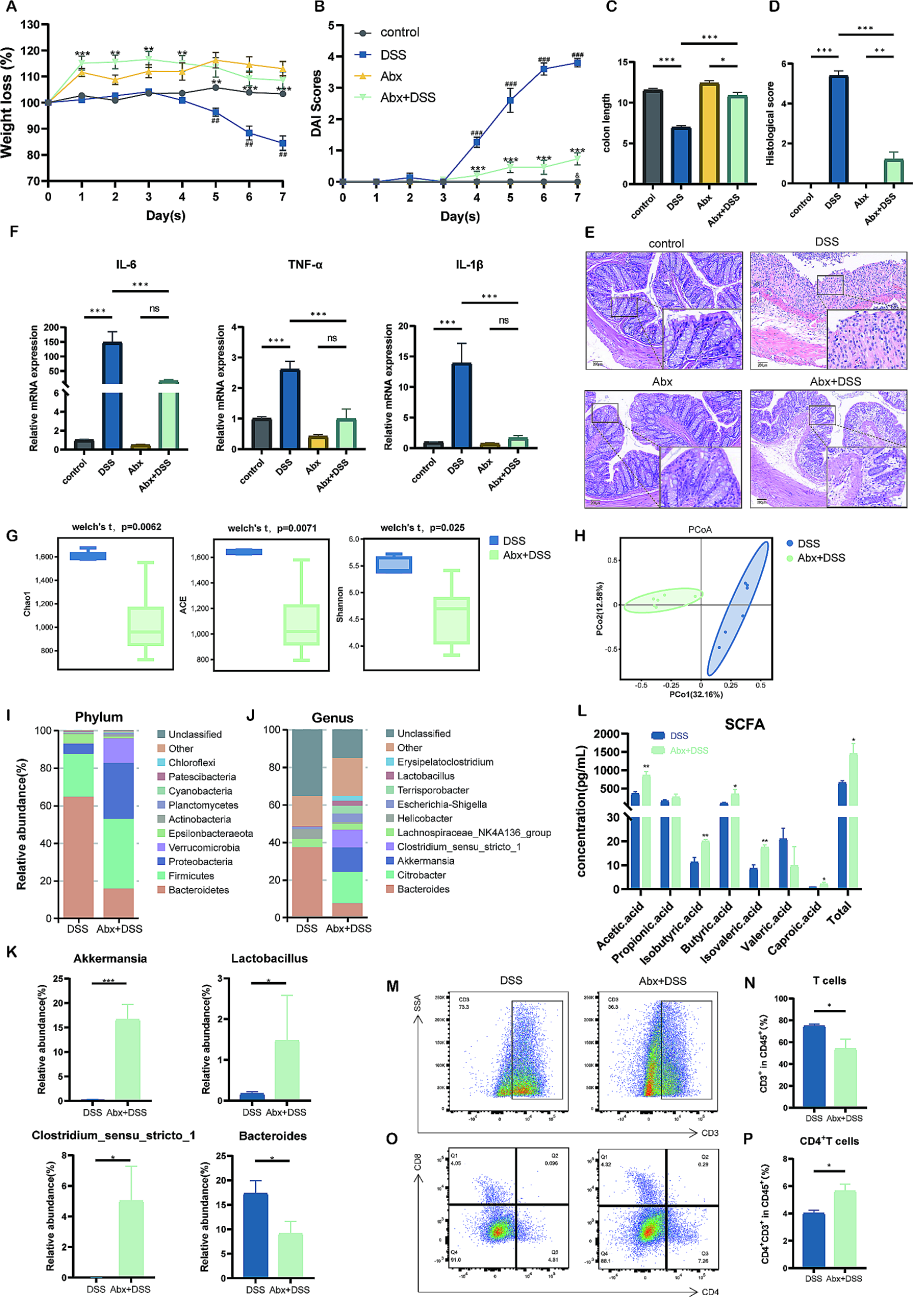
Abx pretreatment ameliorated colitis, regulated dysbiosis, relieved the dysfunction of gut microbiota, and improved colon CD4^+^T cells. The weight loss **(A)** and DAI scores **(B)** in the indicated groups. ^*^P < 0.05, ^**^P < 0.01, and ^***^P < 0.001 compared to the DSS group; ^*##*^*P* < 0.01 and ^*###*^*P* < 0.001 compared to the control group; and ^*&*^*P* < 0.05 compared to the Abx + DSS group. The colon length **(C)** and histological scores **(D)** are depicted with or without Abx pretreatment. **(E)** Representative H&E-staining figures of the colon (10× magnified in the larger frame, 40× magnified in the smaller frame; scale bar − 200 μm). **(F)** IL-6, TNF-α, IL-1β mRNA levels in the indicated groups. **(G)** Alpha-diversity in the indicated groups. **(H)** Unweighted UniFrac based PCoA analysis in the indicated groups. The top 10 dominant taxa among the DSS group and DSS + Abx group, for the phylum level **(I)** and the genus level **(J)**, respectively. **(K)** The relative abundance of *Akkermansia*, *Lactobacillus*, and *Clostridium_sensu_stricto_1*, *Bacteroides*. **(L)** The level of SCFA in feces among groups. The frequency of T cells **(M, N)** and CD4^+^T cells **(O, P)**. These results are indicated as mean ± SEM. ^*^P < 0.05, ^**^P < 0.01, and ^***^P < 0.001 compared to the DSS group. DSS, dextran sodium sulfate; Abx, antibiotic cocktail; Abx + DSS, DSS-induced colitis model with Abx pretreatment; DAI, Disease activity index; and SCFA, short-chain fatty acid

The gut microbiota is closely linked to IBD, both in causing and developing the disease; however, whether pretreating the gut with Abx is more effective in IBD remains controversial. To identify the impact of gut microbiota load on DSS-induced colitis, we built the DSS-induced colitis model with or without Abx pretreatment and evaluated the inflammation of mice. Figure 1A-C indicates that compared with that of the DSS group, weight loss, increased DAI scores, and shortened colon length were improved in the Abx pretreatment group (*P* < 0.001). Subsequently, we conducted a histological analysis of the colon. H&E staining of the distal colon indicated that DSS intervention led to crypt loss, crypt abscess, and excessive infiltration of neutrophils into the lamina propria, all of which are consistent with the pathological characteristics of acute colitis (Fig. 1E). However, compared with the DSS group, DSS-induced mucosal injury and decreased neutrophil infiltration were relieved after Abx pretreatment, which corresponded to lower colonic inflammation (Fig. 1D, *P* < 0.001). Furthermore, the expression of interleukin (IL) -6, tumor necrosis factor (TNF) -α, and IL-1β were all down-regulated after Abx pretreatment (all *P* < 0.001 vs. DSS group; Fig. 1F).

### Abx pretreatment regulated dysbiosis and dysfuncion of gut microbiota and improved colon CD4^+^ T cells

The protective effect of Abx pretreatment on the colitis model was indicated in the aforementioned data. To understand whether Abx pretreatment created a suitable microbiome environment for the gut to improve dysbiosis, we conducted 16 S rRNA sequencing to explore how the structure of gut microbiota changed with or without Abx pretreatment. First, as illustrated in Fig. 1G, compared with the DSS group, Abx pretreatment decreased the alpha-diversity. Specifically, Chao1 (*P* = 0.0062), ACE (*P* = 0.0071), and Shannon (*P* = 0.025) all indicated that species richness and evenness were decreased in the Abx pretreatment group. To observe the change in beta-diversity, we conducted principal coordinate analysis (PCoA). The cluster was distinct between the two groups (PCo1 = 32.16%, PCo2 = 12.58%), as exhibited in Fig. 1H. Subsequently, from the dominant taxa analysis of the phylum level, we found that the Abx pretreatment group was mainly composed of *Firmicutes* (37.10%), *Proteobacteria* (29.78%), and *Bacteroidetes* (15.83%), whereas the DSS group was composed of *Bacteroidetes* (64.64%) and *Firmicutes* (22.84%) (Fig. 1I). In the genus level, we observed that the Abx pretreatment group was mainly from *Citrobacter* (16.83%), *Akkermansia* (12.98%), and *Clostridium_sensu_stricto_1* (9.48%)(Fig. 1J). It is worth noting that the relative abundance of *Akkermansia* (*P* < 0.001), *Lactobacillus* (*P* < 0.05), and *Clostridium_sensu_stricto_1* (*P* < 0.05) was increased, whereas that of *Bacteroides* (*P* < 0.05) was decreased after Abx pretreatment (Fig. 1K). Owing to the beneficial effect of SCFA in IBD patients [29] and the close correlation between metabolites SCFA and the dysfunction of gut microbiota, we utilized gas-phase mass spectrometry to measure the concentration of fecal SCFA. Quantitative analysis led to an elevated level of total SCFA in the Abx pretreatment group (*P* < 0.05), compared with the DSS group (Fig. 1L). Specifically, it was observed that Abx pretreatment could increase the level of acetic acid (*P* < 0.01), isobutyric acid (*P* < 0.01), butyric acid (*P* < 0.05), isovaleric acid (*P* < 0.01), and caproic acid (*P* < 0.05), which might indicate the regulation of gut microbiota dysfunction by Abx pretreatment (Fig. 1L).

In addition, to understand whether Abx pretreatment influenced the immune environment for the gut to enhance immune imbalance, we conducted flow cytometry to explore the changed immune cell type. Interestingly, Abx pretreatment reduced the percentage of colonic T cells compared with only DSS-treated mice (*P* < 0.05; Fig. 1M-N). The CD4^+^ T cell frequency was elevated in the colon from Abx pretreatment compared to only DSS-treated mice (*P* < 0.05; Fig. 1O-P). The CD8^+^ T cell frequency remained unchanged between the DSS group and Abx + DSS group (*P* = 0.0556; Figure S1).

### Abx pretreatment with CBM augmented the anti-inflammatory effect by improving histological injury and reducing pro-inflammatory cytokines

**Fig. 2:**
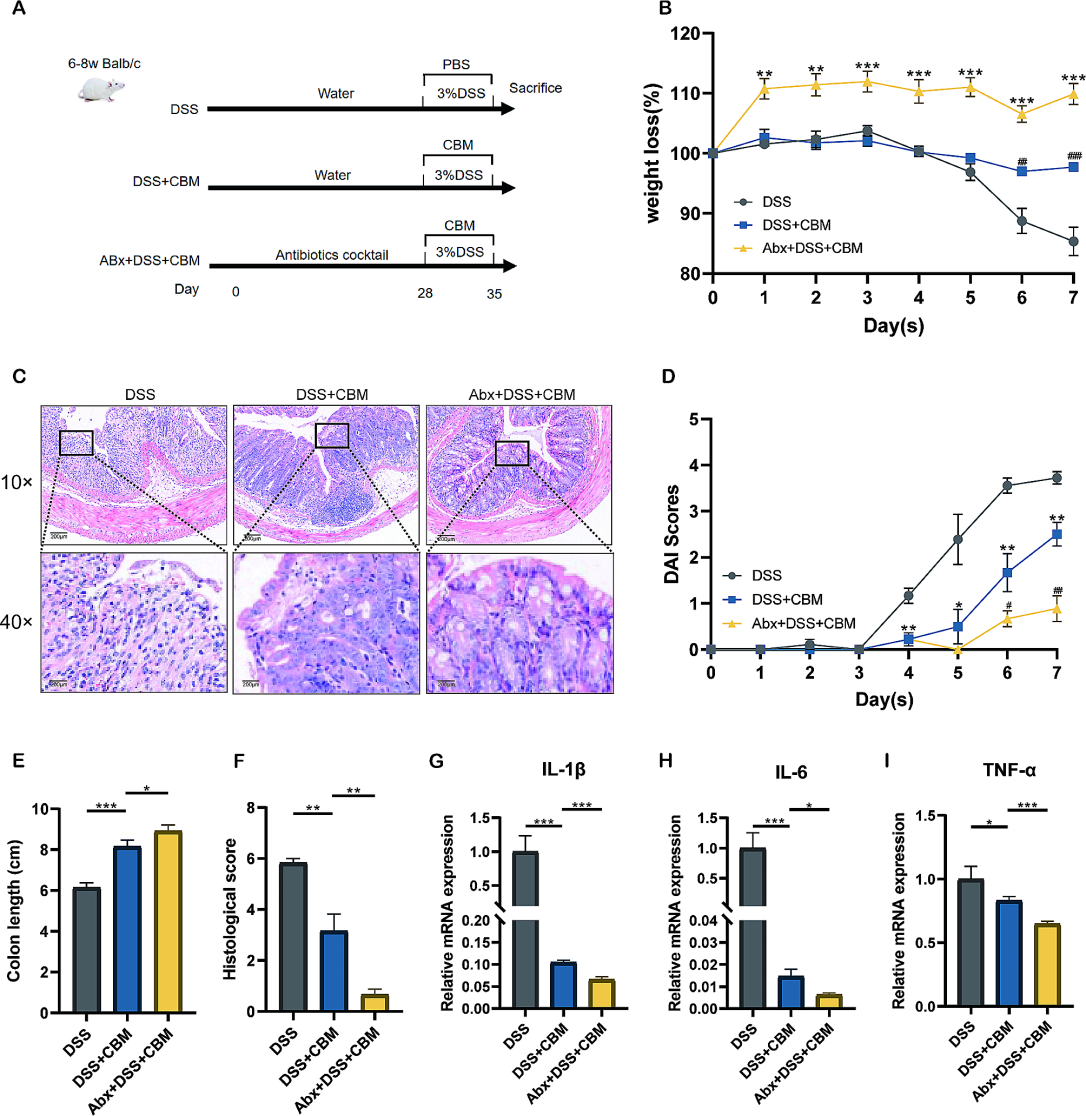
*Clostridium butirycum Miyairi588* (CBM) gavage ameliorated DSS-induced colitis, and its efficacy was enhanced by Abx pretreatment. **(A)** Experimental animal model for acute colitis and pretreatment with Abx cocktail. **(B)** The weight loss in the indicated groups. ^*^P < 0.05, ^**^P < 0.01, and ^***^P < 0.001 compared to the DSS + CBM group; ^*#*^*P* < 0.05, ^*##*^*P* < 0.01, and ^*###*^*P* < 0.001 compared to the DSS group. **(C)** Representative H&E-staining figures of the colon (10× magnification large, 40× magnification small; scale bar – 200 μm). **(D)** DAI of the indicated groups during the experiment. ^*^P < 0.05, ^**^P < 0.01, and ^***^P < 0.001 compared to the DSS + CBM group; ^*#*^*P* < 0.05, ^*##*^*P* < 0.01, and ^*###*^*P* < 0.001 compared to the DSS group. **(E)** Colon length of the indicated groups on day 35. **(F)** Histopathological scores of distal colon tissues. IL-1β **(G)**, IL-6 **(H)**, and TNF-α **(I)** mRNA expression among groups. These results are indicated as mean ± SEM. ^*^P < 0.05, ^**^P < 0.01, and ^***^P < 0.001 compared to the DSS + CBM group. CBM, *Clostridium butirycum Miyairi588*; DAI, Disease activity index; DSS, dextran sodium sulfate; Abx, antibiotic cocktail; DSS + CBM, CBM oral gavage treated colitis mice; and Abx + DSS + CBM, colitis mice treated with CBM combined with Abx pretreatment

To determine the efficacy of CBM in colitis as well as the effect of Abx pretreatment, male SPF Balb/c mice were given normal or Abx-supplemented water for 28 days, and colitis was induced over 7 days with 3% DSS (Fig. 2A). DSS exposure led to the typical symptoms of colitis, which were alleviated after CBM intervention (weight loss at Day 7: *P* < 0.01, colon length: *P* < 0.001, DAI at Day 7: *P* < 0.01; Fig. 2B, E and D). It is worth noting that ABx administration prior to CBM intervention further improved the weight of colitis mice (weight loss at Day 7: *P* < 0.001; Fig. 2B), and also exerted an augmented influence on the protective effects of CBM on DAI (DAI at Day 7: *P* < 0.01; Fig. 2D) and colon shortening (*P* < 0.05; Fig. 2E). Histopathological examination of the distal colon samples indicated that DSS stimulation led to crypt loss, crypt abscess, submucosal edema, mucosal disruption, and the excessive infiltration of neutrophils into the lamina propria, all of which are consistent with the pathological characteristics of acute colitis (Fig. 2C). CBM intervention relieved mucosal injury and decreased neutrophil infiltration, which corresponded to lower colonic inflammation (*P* < 0.01; Fig. 2F). Abx pretreatment further enhanced colon histological structures in the CBM-treated colitis mice compared to that in the Abx + DSS group (*P* < 0.05; Fig. 2F). Moreover, inflammatory cytokines such as IL-1β (*P* < 0.001; Fig. 2G), IL-6 (*P* < 0.001; Fig. 2H), and TNF-α (*P* < 0.05; Fig. 2I) were all decreased after CBM intervention, compared with DSS group. It is worth noting that pretreatment with Abx can also statistically enhance the inhibitory effect of CBM on the expression of these inflammatory cytokines (*P* < 0.001, *P* < 0.05, *P* < 0.001; Fig. 2G-H). In summary, CBM improved inflammation, and Abx pretreatment augmented the therapeutic effect of CBM.

### Abx pretreatment with CBM reinforced the repair of the gut barrier injury

**Fig. 3:**
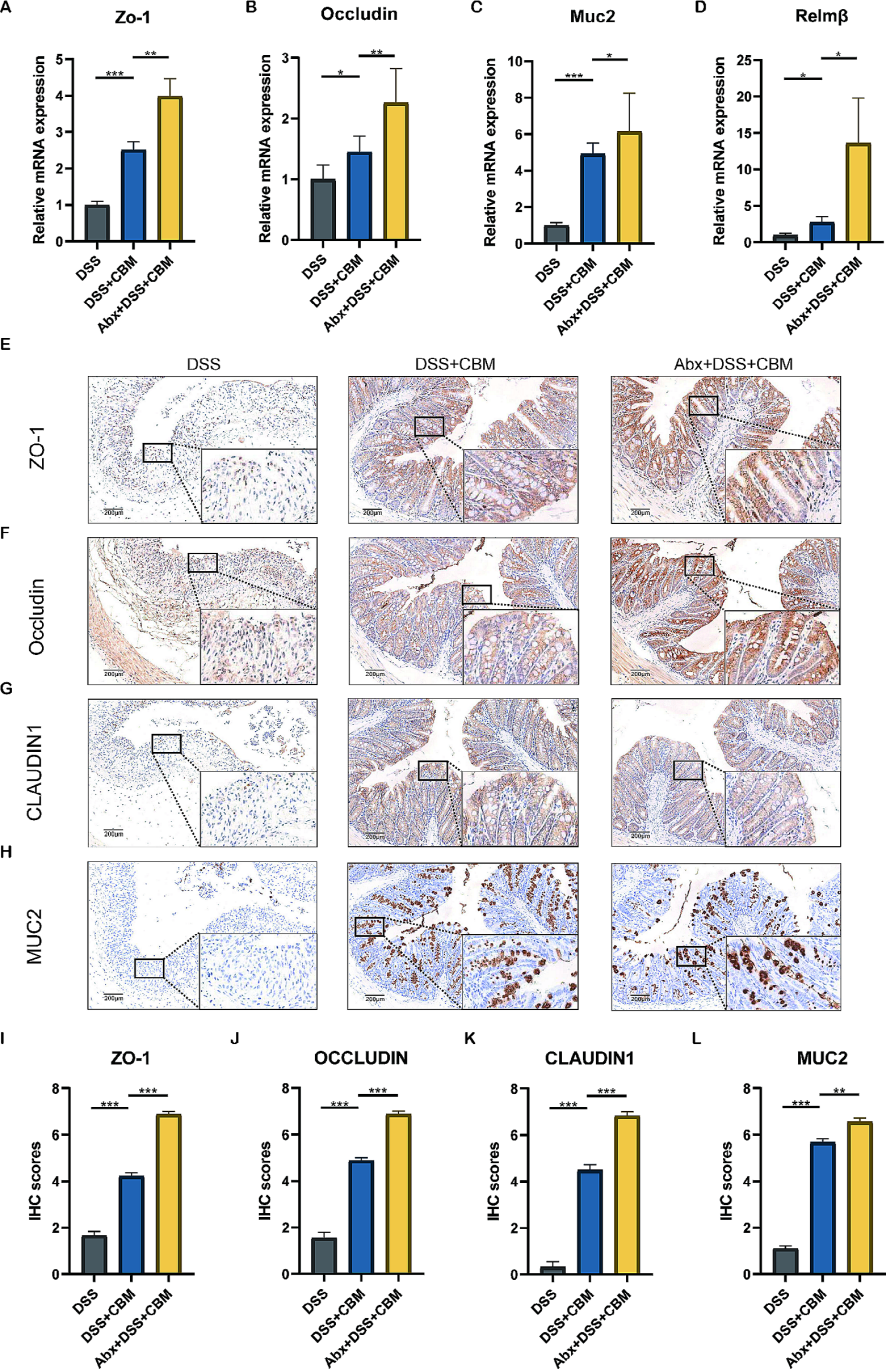
CBM restored the gut barrier damage and Abx pretreatment enhanced the efficacy. Zonula occludens (ZO) -1 **(A)**, Occludin **(B)**, mucin2 (MUC2)**(C)**, and Relmβ **(D)** mRNA levels in the indicated groups. Representative ZO-1 **(E)**, OCCLUDIN **(F)**, CLAUDIN1 **(G)**, and MUC2 **(H)** images, and the related immunohistochemical scores (**I-L**; 10× magnification large, 40× magnification small; scale bar – 200 μm) were presented. These results are expressed as mean ± SEM, *n* = 6. ^*^P < 0.05, ^**^P < 0.01, and ^***^P < 0.001 were compared to the DSS + CBM group. CBM, *Clostridium butirycum Miyairi588*; ZO, Zonula occludens; MUC2, mucin2; DSS, dextran sodium sulfate; Abx, antibiotic cocktail; DSS + CBM, CBM oral gavage treated colitis mice; and Abx + DSS + CBM, colitis mice treated with CBM combined with Abx pretreatment

The mucus layer and tight junction proteins form the physical barrier of the intestinal epithelium. CBM up-regulated the relative mRNA expression of Zo-1 (*P* < 0.001), Occludin (*P* < 0.05), Muc2 (*P* < 0.001), and Relmβ (*P* < 0.05) in the colon compared to that in the DSS group (Fig. 3A-D). Furthermore, Abx pretreatment significantly augmented the CBM-mediated up-regulation of Zo-1 (*P* < 0.01), Occludin (*P* < 0.01), Muc2 (*P* < 0.05), and Relmβ (*P* < 0.05), which indicated that Abx pretreatment has an enhanced influence on the gut barrier protective effect of CBM (Fig. 3A-D).

To observe the change in the gut barrier at the protein level, we conducted immunohistochemical staining of colon tissues. The results indicated that DSS exposure impaired the tight junction of the intestine, which was reversed by CBM treatment (Fig. 3E-H). Interestingly, compared to the DSS group, Abx pretreatment significantly upregulated ZO-1 (*P* < 0.001; Fig. 3I), OCCLUDIN (*P* < 0.001; Fig. 3J), CLAUDIN1 (*P* < 0.001; Fig. 3K), and MUC2 (*P* < 0.01; Fig. 3L), thereby indicating that the Abx pretreatment can enhance intestinal barrier function by altering the gut microbiota. In addition, CBM can increase the level of Occludin (without Abx: *P* < 0.05; with Abx: *P* < 0.001) and Claudin1 (without Abx: *P* < 0.05; with Abx: *P* < 0.001) detected by western blot with or without Abx pretreatment (Figure S3A-B, Figure S4-S5). In summary, CBM restored the intestinal barrier in colitis mice, and its protective effect was enhanced by Abx pretreatment.

**Effects of CBM on dysbiosis and the dysfunction of microbiota in the gut of Abx-pretreatment mice**.

**Fig. 4:**
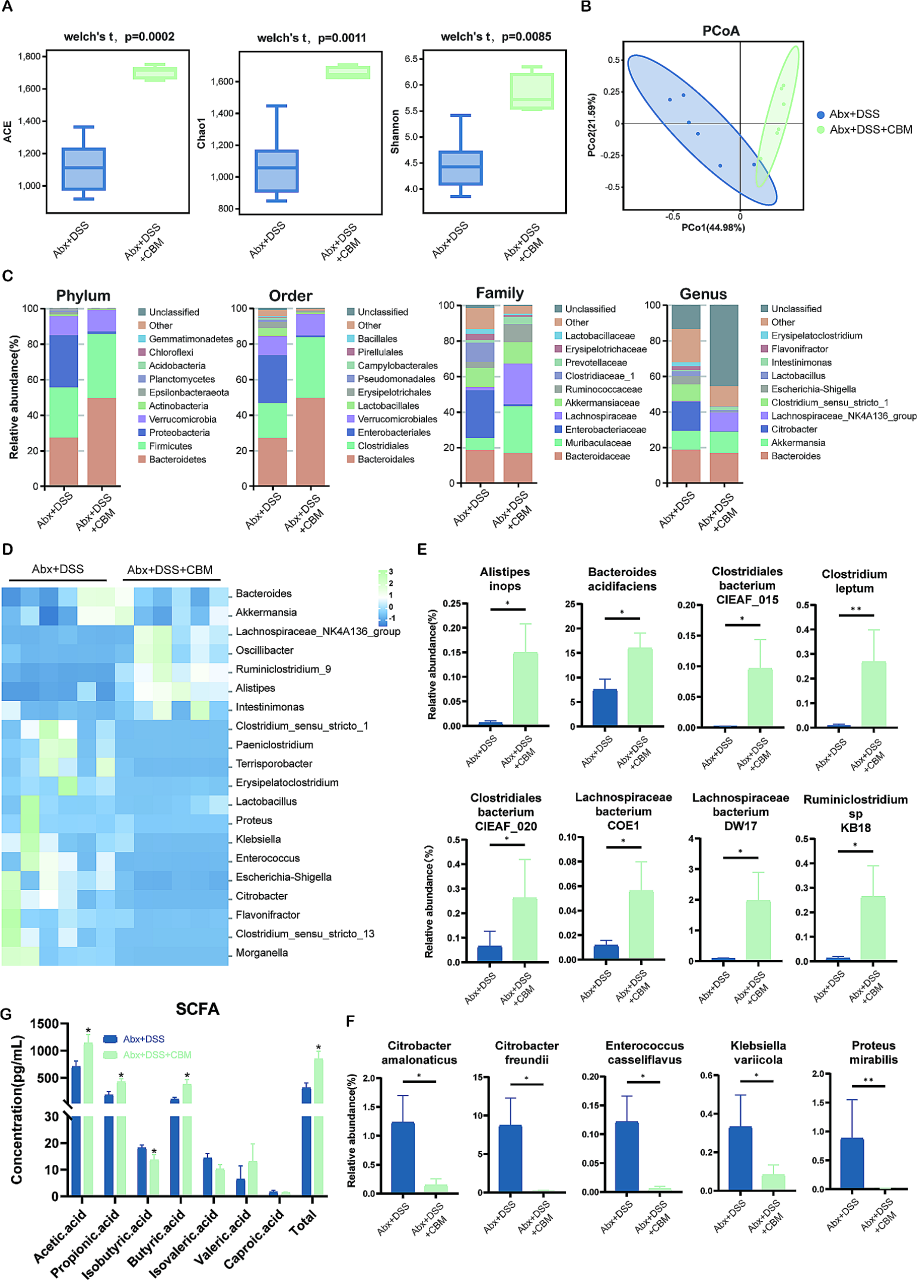
CBM reversed gut dysbiosis and SCFA metabolism after pretreatment with Abx. **(A)** The alpha-diversity in the Abx pretreated colitis with or without CBM intervention. **(B)** Unweighted UniFrac based PCoA analysis among groups. **(C)** The top 10 dominant bacteria taxa in the indicated groups at the level of phylum, order, family, and genus. **(D)** Heatmap of genus taxa with a relative abundance greater than 1% in the indicated group. **(E)** The up-regulated species in the Abx pretreated colitis with CBM intervention. **(F)** The down-regulated species in the Abx pretreated colitis with CBM intervention. **(G)** Fecal SCFA concentration among groups. These results are expressed as mean ± SEM. ^*^P < 0.05, ^**^P < 0.01, and ^***^P < 0.001 compared to the Abx + DSS group. CBM, *Clostridium butirycum Miyairi588*; DSS, dextran sodium sulfate; Abx, antibiotic cocktail; Abx + DSS, DSS-induced colitis model with Abx pretreatment; Abx + DSS + CBM, colitis mice treated with CBM combined with Abx pretreatment; and SCFA, short chain fatty acid

We analyzed the fecal microbiota in the different groups using 16 S rRNA sequencing. The previous studies have indicated that Abx pretreatment removed most bacteria from the intestinal tract, which may be useful for analyzing individual bacteria [13]. The ACE index, Chao1 index, and Shannon index, which indicate the α diversity of gut microbiota, were higher after CBM intervention (*P* = 0.0002, *P* = 0.0011, *P* = 0.0085; Fig. 4A). PCoA analysis further indicated that the two Abx pretreatment were well-separated after CBM intervention (PCo1 = 44.98%, PCo2 = 21.59%; Fig. 4B), indicating that the β diversity was altered in colitis mice.

The top 10 most abundant taxa in each group represent the dominant taxa composition (Fig. 4C). First, the taxa were mainly from *Bacteroidetes* (49.50%), *Firmicutes* (36.15%), and *Proteobacteria* (12.04%) after CBM intervention, whereas the Abx + DSS group was mainly from *Bacteroidetes* (27.31%), *Proteobacteria* (29.22%), and *Firmicutes* (28.28%) at the phylum level. Subsequently, at the order level, the taxa were mainly from *Bacteroidales* (49.48%), *Clostridiales* (34.22%), and *Verrucomicrobiales* (12.03%) after CBM intervention, whereas the Abx + DSS group was mainly from *Bacteroidales* (27.01%), *Enterobacteriales* (26.78%), and *Clostridiales* (19.72%). Interestingly, at the family level, the taxa were mainly from *Bacteroidaceae* (16.76%), *Muribaculaceae* (26.54%), and *Lachnospiraceae* (22.97%) after CBM intervention, whereas the Abx + DSS group was mainly from *Bacteroidaceae* (18.59%), *Enterobacteriaceae* (26.78%), and *Akkermansiaceae* (10.66%). For the genus level, the taxa were mainly from the *Bacteroides* (16.76%), *Akkermansia* (12.02%), and *Lachnospiraceae_NK4A136_group* (10.61%) after CBM intervention, whereas the Abx + DSS group was mainly from *Bacteroides* (18.59%), *Citrobacter* (16.42%), and *Akkermansia* (10.66%). Interestingly, the DSS group also exhibited different relative abundance at the aforementioned levels (Table S2).

Furthermore, the analysis of bacterial genus with relative abundance > 1% by heatmap indicated that some genus were enriched after CBM intervention (Fig. 4D), including *Lachnospiraceae_NK4A136_group*, *Oscillibacter*, *Ruminiclostridium_9*, *Alistipes*, and *Intestinimonas*. To clarify the statistically modified species, we analyzed the top 30 dominant taxa among the Abx pretreated groups with or without CBM intervention. Data indicated that CBM can up-regulate the taxa of *Alistipes inops* (*P* < 0.05), *Bacteroides acidifaciens* (*P* < 0.05), *Clostridiales bacterium CIEAF_015* (*P* < 0.05), *Clostridium leptum* (*P* < 0.01), *Clostridiales bacterium CIEAF_020* (*P* < 0.05), *Lachnospiraceae bacterium COE1* (*P* < 0.05), *Lachnospiraceae bacteria DW17* (*P* < 0.05), and *Ruminiclostridium sp KB18* (*P* < 0.05) (Fig. 4E), while decreasing that of *Citrobacter amalonaticus* (*P* < 0.05), *Citrobacter freundii* (*P* < 0.05), *Enterococcus casseliflavus* (*P* < 0.05), *Klebsiella variicola* (*P* < 0.05), and *Proteus mirabilis* (*P* < 0.01)(Fig. 4F). Interestingly, most of these up-regulated taxa belong to the SCFA-producing *Lachnospiraceae* family, *Ruminococcus* family, and *Clostridium* family.

It is worth restating that CBM intervention increased the taxa associated with SCFA, including *Muribaculaceae*, *Lachnospiraceae*, and *Ruminococcus* (the family level; Fig. 4C). To clarify the change of SCFA in the feces of mice after CBM intervention, gas-phase mass spectrometry was conducted, and led to an elevated level of total SCFA in the CBM intervention group (*P* < 0.05; Fig. 4G). Specifically, CBM intervention could increase the level of acetic acid (*P* < 0.05), propionic acid (*P* < 0.05), isobutyric acid (*P* < 0.05), and butyric acid (*P* < 0.05), which might indicate the regulation of gut microbiota dysfunction by CBM combined with Abx pretreatment. In addition, the DSS group also exhibited a different concentration of SCFA compared with the Abx + DSS and Abx + DSS + CBM groups (Table S2).

In summary, the beneficial taxa were increased, and potential pathogens were decreased; moreover, the SCFA metabolism of gut microbiota were improved after CBM intervention combined with Abx pretreatment. It is reasonable to assume that CBM intervention combined with Abx pretreatment restored the gut microbiota structure and dysfunction of colitis mice.

### Effects of CBM on inflammatory cytokines and CD4^+^T cells in abx-pretreatment mice

**Fig. 5:**
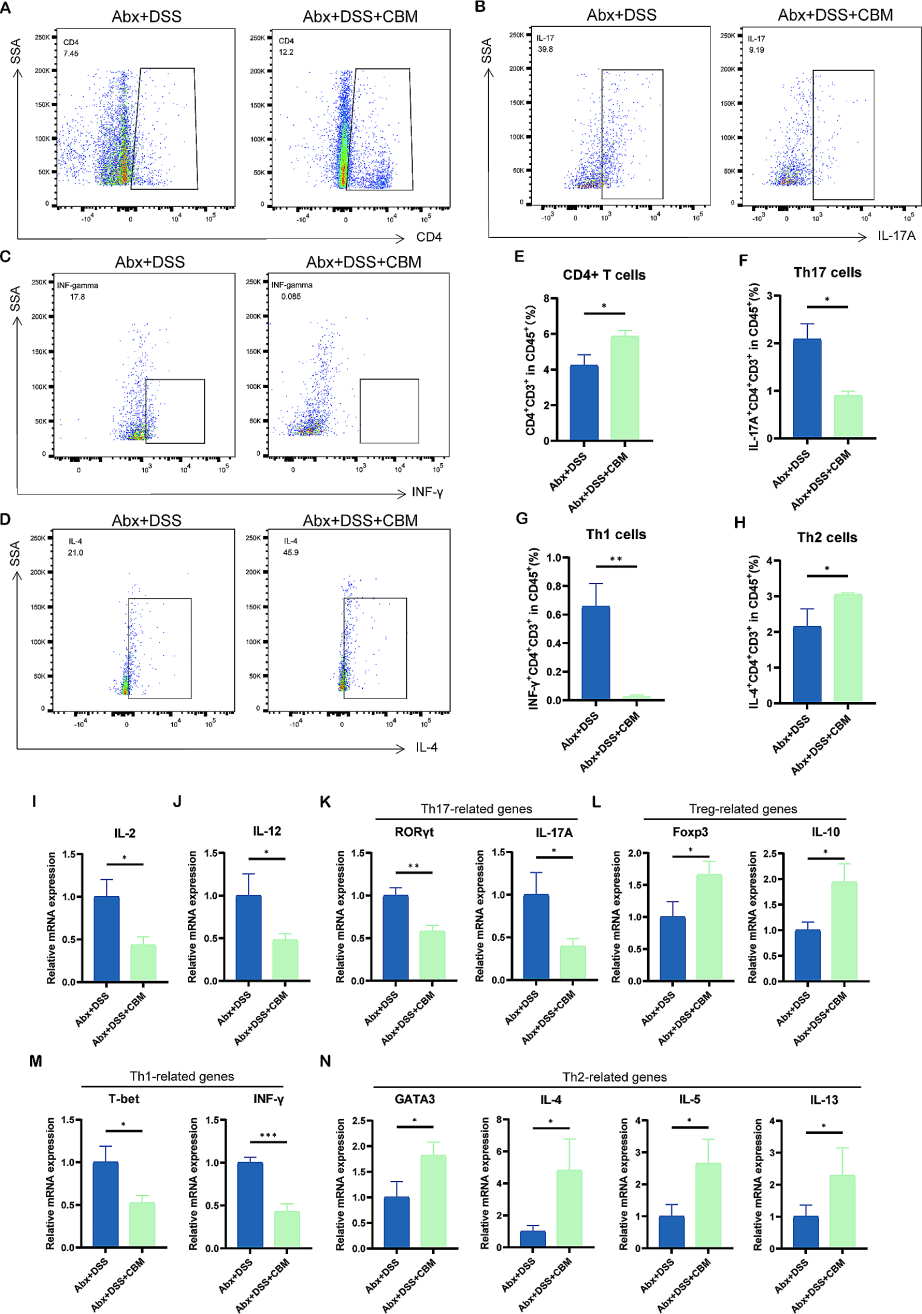
CBM regulated the immune response in colitis mouse after pretreatment with Abx. The frequency of CD4^+^ T cells **(A, E)**, CD4^+^ IL-17 A^+^ Th17 cells **(B, F)**, CD4^+^ IFN-γ^+^ Th1 cells **(C, G)**, and CD4^+^ IL-4^+^ Th2 cells **(D, H)**. **(I-J)** The relative expression of IL-2, IL-12 in colon tissues. **(K)** RORγt, IL-17 A expression of Th17 cells-related cytokines, and transcription factors in the mice of Abx pretreated with or without CBM intervention. **(L)** Foxp3, IL-10 expression of Tregs-related cytokines, and transcription factors in the indicated groups. **(M)** T-bet, INF-γ expression of Th1 cells-related cytokines, and transcription factors in the indicated groups. **(N)** GATA3, IL-4, IL-5, IL-13 expression of Th2 cells-related cytokines and transcription factors among groups. These results are expressed as mean ± SEM. ^*^P < 0.05, ^**^P < 0.01, and ^***^P < 0.001 compared to the Abx + DSS group. CBM, *Clostridium butirycum Miyairi588*; DSS, dextran sodium sulfate; Abx, antibiotic cocktail; Abx + DSS, DSS-induced colitis model with Abx pretreatment; and Abx + DSS + CBM, colitis mice treated with CBM combined with Abx pretreatment

To understand whether the CD4^+^T cells’ immune response was regulated by CBM intervention combined with Abx pretreatment, the frequency of CD4^+^ T cells and their subtypes was determined. The frequency of CD4^+^T cells was upregulated after CBM treatment (*P* < 0.05; Fig. 5A and E). Percentage of helper T (Th) 17 cells (IL-17 A^+^CD4^+^, *P* < 0.05; Fig. 5B and F) and Th1 cells (IFN-γ^+^CD4^+^, *P* < 0.01; Fig. 5C and G) for CD4 + T cell subtypes, which were considered as pro-inflammatory immune cells, were decreased after CBM intervention, whereas the frequency of anti-inflammatory Th2 cells (IL-4^+^CD4^+^, *P* < 0.05; Fig. 5D and H) was elevated. However, CBM intervention had a decreased effect on Tregs with Abx pretreatment (Foxp3^+^CD4^+^, *P* < 0.01; Figure S2).

Cytokines can stimulate and be released by immune cells, and transcription factors can indicate the condition of functional immune cells; therefore, a panel of cytokines and transcription factors in the colon were detected. To evaluate how the CD4^+^T immune response was regulated by CBM intervention combined with Abx pretreatment, the relative expression of cytokines and transcription factors were measured. Interestingly, pro-inflammatory cytokines related to innate immune including IL-2 (*P* < 0.05; Fig. 5I), and IL-12 (*P* < 0.05; Fig. 5J) were decreased by CBM intervention. It is worth noting that CBM reduced the relative expression of transcription factor RORγt and cytokine IL-17a with pro-inflammatory Th17 cells (*P* < 0.01, *P* < 0.05; Fig. 5K). Moreover, the relative expression of T-bet and INF-γ with pro-inflammatory Th1 cells was also decreased in CBM-treated mice (*P* < 0.05, *P* < 0.001; Fig. 5M). Additionally, the relative expression of transcription factor Foxp3 and cytokine IL-10 with anti-inflammatory Tregs were also upregulated in CBM-treated mice (*P* < 0.05, *P* < 0.05; Fig. 5L), as well as the transcription factor GATA3 and cytokines IL-14, IL-5, and IL-13 with anti-inflammatory Th2 cells (all *P* < 0.05; Fig. 5N). In summary, CBM enhanced intestinal immune homeostasis after Abx pretreatment by regulating CD4^+^T cells and their subtypes.

## Discussion

It is proposed that IBD is caused by several factors such as environment, genetics, immunity, and gut microbiota, which increases the difficulty of analyzing IBD in humans. Therefore, the DSS-induced colitis mice model is mainly utilized to analyze the etiology, pathogenesis, and clinical treatment of IBD—especially the correlation between UC and intestinal immunity and microbiota. Herein, the colitis mice manifested diarrhea, bloody stool, weight loss, and decreased activity, symptoms which are quite similar to those of human UC; the aforementioned observation indicates that the colitis model has been successfully constructed. Furthermore, the colitis mice pretreated with Abx had less inflammation compared to the untreated colitis mice, which is consistent with a previous study that exhibited remission of acute UC symptoms in patients receiving Abx pretreatment [30]. A randomized controlled trial further indicated that Abx reduced disease activity in children with acute severe colitis on day 5 of administration [31]. Abx can potentially eliminate some pathogens and conditional pathogens from the intestinal tract, thereby alleviating the symptoms of colitis. However, some studies have associated Abx treatment with increased gut inflammation [32]. The results indicated that Abx pretreatment can effectively relieve intestinal inflammation on improving weight, colon shorting, and histological injury, compared to DSS-induced colitis mice. Meanwhile, the dysbiosis and dysfunction of gut microbiota were enhanced with Abx pretreatment, especially on SCFA metabolism. These indicated that Abx pretreatment regulates the imbalance of gut microbiota and might be useful for further microbiota-based therapy. In addition, CD4^+^ T cell differentiation profiles often associated with the improvement in DSS-induced colitis [33]. The results indicated that Abx pretreatment could regulate the imbalance of T cells’ immune response, especially on CD4^+^ T cells. These observations potentially indicate the immune regulation effect of Abx pretreatment on colitis. The preceding results indicated that Abx pretreatment might create a suitable condition for microbiota-based therapy.

The incidence of IBD is increasing globally, and the drugs currently utilized to alleviate its symptoms are largely ineffective and fraught with adverse effects. Microbiota-based interventions have been considerably developed in recent years, especially probiotic treatments. For instance, some probiotics have proved to be therapeutic against non-alcoholic fatty liver disease (NAFLD) and dysbiosis-induced diarrhea [7, 34]. Herein, CBM can relieve the intestinal inflammation on weight loss, gut barrier injury, and histological injury of colitis mice effectively, which is consistent with previous studies [35, 36].

Owing to the rapid advances in microbiota-based interventions, identifying the most optimal conditions for their efficacy has become a necessity. Abx pretreatment was commonly utilized to simulate germ-free mouse models; few studies have focused on its effect on probiotic interventions. A study has revealed that Abx treatment before FMT can enhance the curative effect of the latter on UC patients [17]. In addition, a randomized trial of ulcerative colitis indicated the stain profile, and the results revealed that antibiotic pretreatment can be useful prior to FMT [37]. However, reports are indicating that Abx can aggravate the inflammatory symptoms of colitis in mice [32]. The results indicated that the enhanced inflammation and gut barrier of colitis by CBM intervention were both strengthened after Abx pretreatment. This indicates that Abx pretreatment can enhance the efficacy of CBM intervention, which may be related to the modulating effect of Abx pretreatment on gut imbalance.

The intestinal barrier is the first line of defense and maintains the homeostasis that protects the host against pathogens, while promoting the colonization of probiotics. The mucosa is the outermost part of the intestinal barrier and comprises two layers of mucus attached to epithelial cells. Glycosylated MUC2 protein forms mucus secreted by goblet cells in the gut [38, 39]. The deletion of the MUC2 gene leads to the development of spontaneous colitis in a mouse model [40]. Relmβ, another goblet cell-specific protein, transfers intestinal lumen antigens to DCs and promotes the Th2 response [41, 42]. In the acute stage of UC, the colonic mucus layer becomes thinner due to a reduction in the number of goblet cells and the production of MUC2 [43]. Although MUC2 expression is normal in CD patients, there are structural anomalies that obliterate its protective effect on the intestinal epithelium [44]. The intestinal epithelial cells and intercellular tight junction proteins, including Zo, occludin, and claudin family proteins, also constitute a major part of the mechanical intestinal barrier. The expression levels of several tight junction proteins are altered in IBD patients [45]. Additionally, decreased tight junction proteins are common in UC and CD patients, which corresponds to epithelial cell loss and gut barrier damage [46–48].

There is ample evidence for a close relationship between gut bacteria and the intestinal barrier. Studies indicate that the abundance of mucus-degrading bacteria is increased in IBD patients [49, 50]. The mucus production can be restored by probiotics such as *B. Thetaiotamicron* and *Faecalibacterium prausnitzii*, which increase goblet cell differentiation and glycosylation gene expression [51, 52]. . *Lactobacillus* upregulates MUC3 in human intestinal epithelial cells and promotes MUC2 production and secretion [53, 54]. Similarly, CBM also alleviated intestinal barrier damage in dysbiosis [7]. Consistent with the preceding results, we found that CBM restored the intestinal barrier in colitis by repairing mucin and tight junction injuries, and the number of goblet cells. In addition, Abx pretreatment enhanced the anti-inflammatory and enhanced barrier injury of CBM, which is occasioned by the elimination of some conditional pathogens by Abx.

As for gut microbiota, CBM could up-regulate the alpha-diversity of mice with colitis, which was indicative of the gut microbiota’s increased richness, compared with only the Abx pretreatment group. In addition, CBM also enhanced the gut microbiota’s β-diversity. Mice with DSS-induced colitis had a lower abundance of *Fimicutes*, which was increased by CBM intervention. In a previous study, *Proteobacteria* was the main phylum in the intestine of mice pretreated with Abx [13]. However, CBM could decrease the abundance of both *Proteobacteria*, *Fusobacteria*, and *Enterobacteriaceae*, which were considered potential pathogens, and could especially increase that of the SCFA-producing taxa. *Citrobacter_freundii* leads to bacterial meningitis by invading and propagating microvascular endothelial cells in the brain [55], and can also aggravate colitis in mice [56]. *Enterococcus_casseliflavus* is resistant to metronidazole, which is related to the occurrence of most infectious diseases [57]. In addition, several beneficial taxa such as *Clostridium_bacterium_CIEAF_015*, *Clostridiales_bacterium_CIEAF_020*, *Clostridium_leptum*, *Lachnospiraceae_bacterium_COE1*, *Lachnospiraceae_NK4A136_group*, and *Lachnospiraceae_bacterium_DW17* were increased by CBM with Abx pretreatment. Moreover, one of the bacterial metabolites, SCFAs, is derived via the bacterial fermentation of indigestible fibers and related to the regulation of gut inflammation. In particular, IBD patients have a lower abundance of butyric acid-producing bacteria compared to healthy controls [58]. The concentration of total SCFAs in feces increased after CBM intervention. Similar trends were observed with some individual SCFAs such as acetic acid, propionic acid, isobutyric acid, and butyric acid. We speculate that Abx pretreatment provides a favorable environment for the growth of beneficial bacteria and that CBM can effect a more effective role in regulating dysbiosis and metabolic imbalance in the gut. In addition, the elimination of most gut bacteria by Abx likely enabled CBM to grow rapidly and colonize the intestinal tract, which in turn augmented CBM’s anti-inflammatory effects.

The gut microbiota exerts a crucial impact on the host’s immune response. The study found that the probiotic restored the intestinal barrier in mice with gut dysbiosis by activating the IL-17a-producing γδT cells [7]. Moreover, CBM can effectively relieve diarrhea occasioned by *Clostridium difficile* infection by regulating the infiltration of pro-inflammatory immune cells in the colon [59]. The study [60] indicated that *C. butyricum* alleviated TNBS-induced colitis in IBS mice by decreasing the number of DCs. The inflammatory cytokines and CD4^+^ T cells were regulated by CBM intervention with Abx pretreatment, which might indicate the beneficial immune response was activated after the probiotic intervention. CBM regulated the imbalance of inflammatory cells after pretreatment with Abx, which is known to limit inflammation by secreting specific cytokines [61]. In summary, CBM can regulate the mucosal immune response following Abx pretreatment by balancing the immune cell populations and the cytokine profile.

The study highlighted the effect of Abx in colitis and CBM treatment, which might provide theoretical evidence for microbiota-based therapy, such as CBM intervention. However, this study exhibits some limitations that ought to be considered. For instance, we did not analyze the ability of CBM to colonize with or without Abx pretreatment. Moreover, further research into the underlying mechanisms and clinical translation is necessary.

## Conclusions

Abx can relieve the inflammation of colitis and regulate the dysbiosis and dysfunction of gut microbiota and T cell response in the colon. In addition, Abx pretreatment reinforced the function of CBM in ameliorating inflammation and barrier damage by increasing beneficial taxa, eliminating pathogens, and inducing protective Th2 cell response. In summary, the study reveals a link between Abx pretreatment, microbiota, and immune response changes in colitis, which opens novel research possibilities and provides an experimental basis for Abx treatment prior to FMT or probiotics administration.

### Electronic supplementary material

Below is the link to the electronic supplementary material.

Supplementary Material 1

Supplementary Material 2

Supplementary Material 3

Supplementary Material 4

Supplementary Material 5

Supplementary Material 6

## Data Availability

The 16 S rRNA sequencing data have been submitted into a sequence read archive (SRA) database (The accession number is PRJNA 796185).

## Abbreviations

DSS: Dextran sulfate sodium
IBD: Inflammatory bowel disease
Abx: Antibiotic cocktail
CBM: *Clostridium butyricum Miyairi588*
UC: Ulcerative colitis
SCFAs: Short-chain fatty acids
CD: Crohn’s disease
FMT: Fecal microbiota transplantation
ILCs: Intrinsic lipid cells
DCs: Dendritic cells
DAI: Disease activity index
ZO: Zonula occludens
MUC2: Mucin2
