# Supplementary figures and images for: Pretreatment with an antibiotics cocktail enhances the protective effect of probiotics by regulating SCFA metabolism and Th1/Th2/Th17 cell immune responses
Source: BMC Microbiol. 2024 Mar 18;24:91. doi: 10.1186/s12866-024-03251-2 (PMC10946100; doi:10.1186/s12866-024-03251-2)

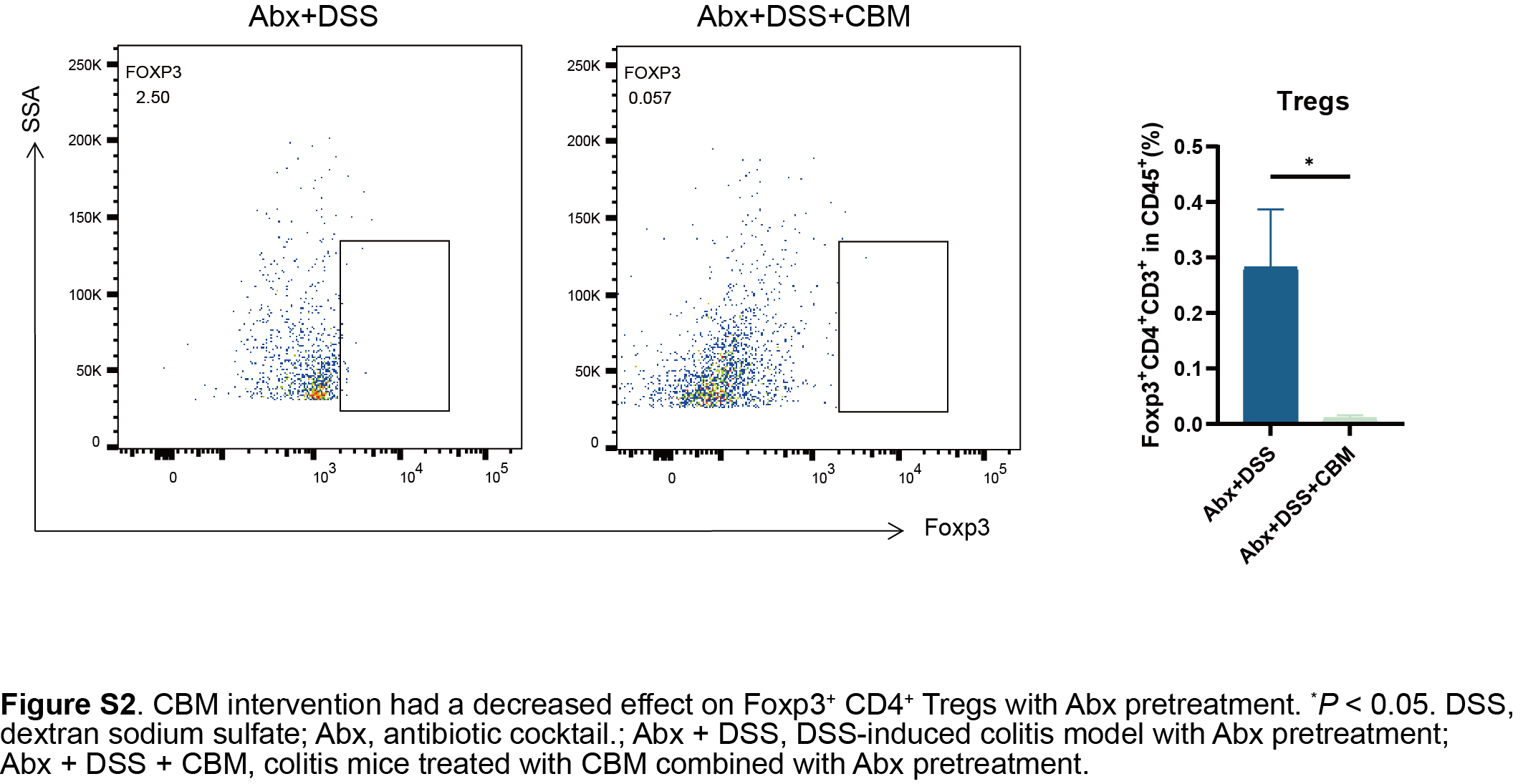

Supplement: Supplementary file 1 — Supplementary Material 1 [file 12866_2024_3251_MOESM1_ESM.png]

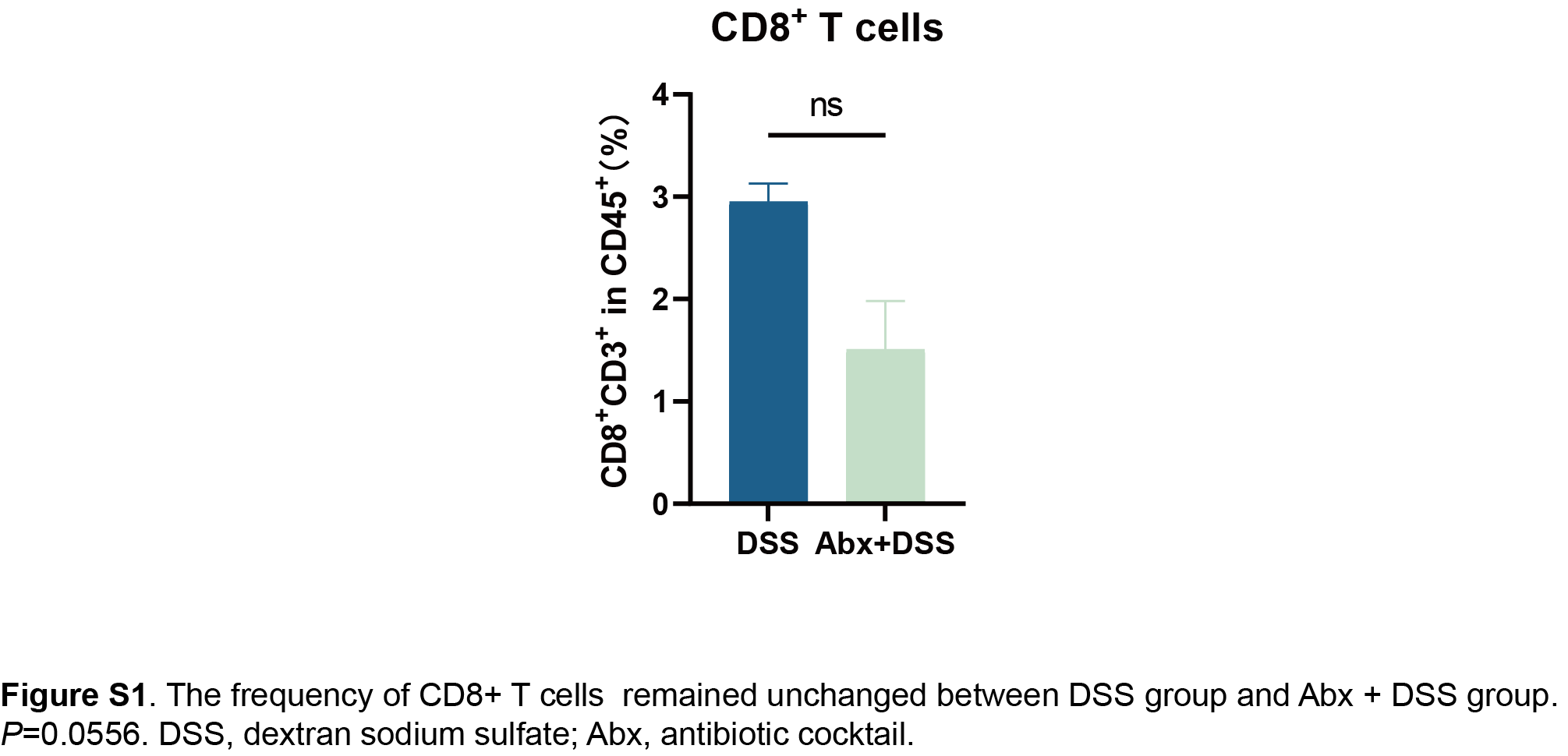

Supplement: Supplementary file 2 — Supplementary Material 2 [file 12866_2024_3251_MOESM2_ESM.png]

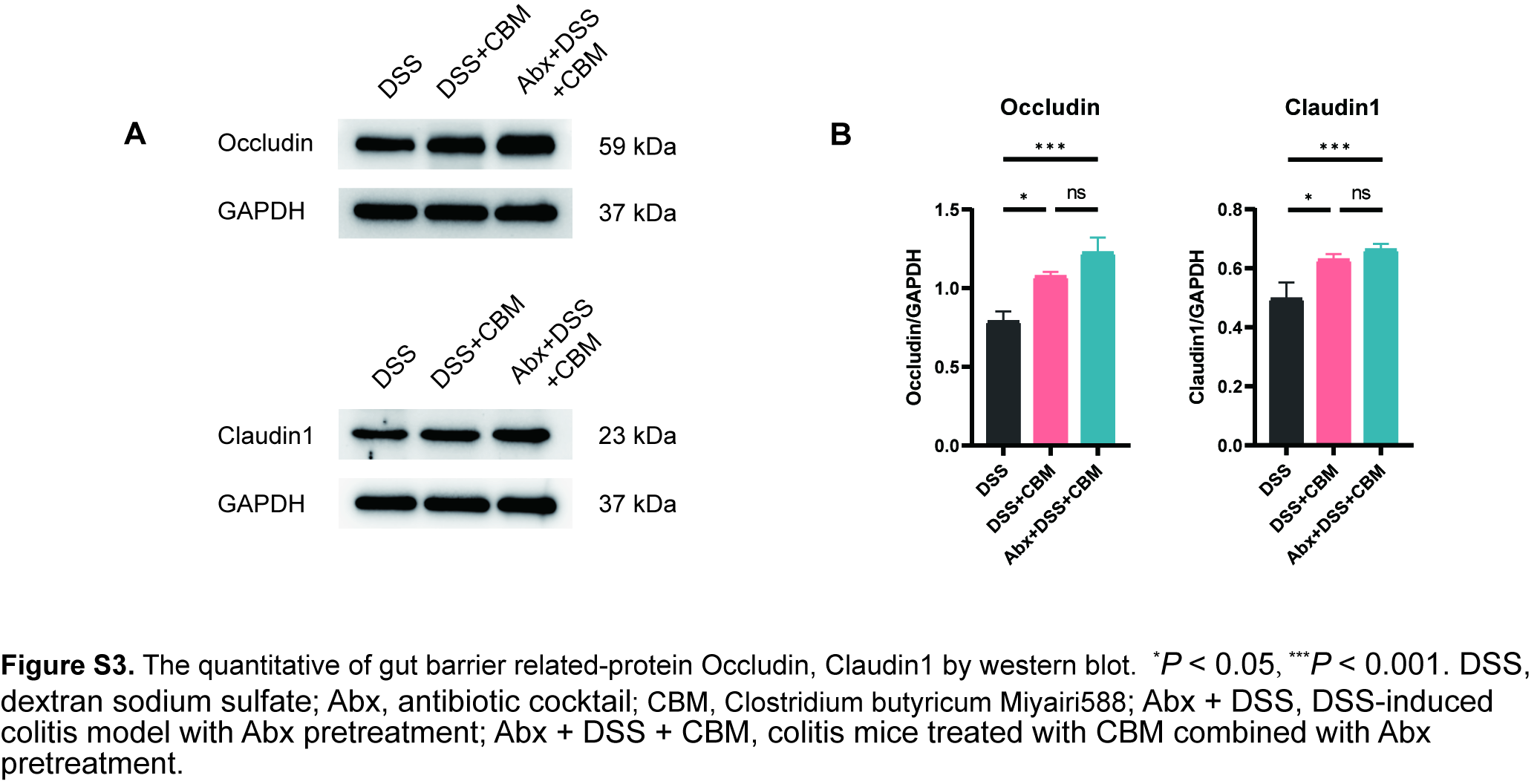

Supplement: Supplementary file 4 — Supplementary Material 4 [file 12866_2024_3251_MOESM4_ESM.tif]
