# Supplementary material for: Pretreatment with an antibiotics cocktail enhances the protective effect of probiotics by regulating SCFA metabolism and Th1/Th2/Th17 cell immune responses
Source: BMC Microbiol. 2024 Mar 18;24:91. doi: 10.1186/s12866-024-03251-2 (PMC10946100; doi:10.1186/s12866-024-03251-2)
Supplement: Supplementary file 3 — Supplementary Material 3 [file 12866_2024_3251_MOESM3_ESM.docx]

Table S1

Sequences of primers

| Gene | Sequence |
| --- | --- |
| *Zo-1* | *Forward: 5’- CAAAGCCCACCAAGGTCAC-3’* |
|  | *Reverse: 5’- TCTCTTTCCGAGGCATTAGCA-3’* |
| *Occludin* | *Forward: 5’-GTGGAGTTGCGGGAGAGC-3’* |
|  | *Reverse: 5’- TCCCAAGATAAGCGAACCTGCC-3’* |
| *Muc2* | *Forward: 5’-CCCAGAAGGGACTGTGTATG-3’* |
|  | *Reverse: 5’- TGCAGACACACTGCTCACA-3’* |
| *Relmβ* | *Forward: 5’-AGCTCCAGGAGGCTGACTTT-3’* |
|  | *Reverse: 5’- CAGGAGATCGTCTTAGGCTC-3’* |
| *Tnfα* | *Forward: 5’-TTAGAAAGGGGATTATGGCTCA-3’* |
|  | *Reverse: 5’- ACTCTCCCTTTGCAGAACTCAG-3’* |
| *Il-6* | *Forward: 5’-GTTGCCTTCTTGGGACTGATG-3’* |
|  | *Reverse: 5’- ATTGCCATTGCACAACTCTTT-3’* |
| *Il-1β* | *Forward: 5’-AGAGCATCCAGCTTCAAATCTC-3’* |
|  | *Reverse: 5’- CAGTTGTCTAATGGGAACGTCA-3’* |
| *Il-17a* | *Forward: 5’-CCTCAAAGCTCAGCGTGTCC-3’* |
|  | *Reverse: 5’- GAGCTCACTTTTGCGCCAAG-3’* |
| *Il-2* | *Forward: 5’-GCGGCATGTTCTGGATTTGACTC-3’* |
|  | *Reverse: 5’- CCACCACAGTTGCTGACTCATC-3’* |
| *Il-4* | *Forward: 5’-TCGGCATTTTGAACGAGGTC-3’* |
|  | *Reverse: 5’- GAAAAGCCCGAAAGAGTCTC-3’* |
| *Il-10* | *Forward: 5’-CGGGAAGACAATAACTGCACCC-3’* |
|  | *Reverse: 5’- CGGTTAGCAGTATGTTGTCCAGC-3’* |
| *Ifnγ* | *Forward: 5’-CATCTTGGCTTTGCAGCTCT-3’* |
|  | *Reverse: 5’- TCTTCCACATCTATGCCACTTG-3’* |
| *Il-5* | *Forward: 5’-CACCAGCTATGCATTGGAGA-3’* |
|  | *Reverse: 5’- TTTGGCGGTCAATGTATTTCT-3’* |
| *Il-13* | *Forward: 5’-AACGGCAGCATGGTATGGAGTG-3’* |
|  | *Reverse: 5’- TGGGTCCTGTAGATGGCATTGC-3’* |
| *Il-12* | *Forward: 5’- ACGAGAGTTGCCTGGCTACTAG-3’* |
|  | *Reverse: 5’- CCTCATAGATGCTACCAAGGCAC-3’* |
| *Rorγt* | *Forward: 5’-GTGGAGTTTGCCAAGCGGCTTT-3’* |
|  | *Reverse: 5’- CCTGCACATTCTGACTAGGACG-3’* |
| *Foxp3* | *Forward: 5’- CCTGGTTGTGAGAAGGTCTTCG-3’* |
|  | *Reverse: 5’- TGCTCCAGAGACTGCACCACTT-3’* |
| *Tbet* | *Forward: 5’- CCACCTGTTGTGGTCCAAGTTC -3’* |
|  | *Reverse: 5’- CCACAAACATCCTGTAATGGCTTG -3’* |
| *Gata3* | *Forward: 5’- CCTCTGGAGGAGGAACGCTAAT-3’* |
|  | *Reverse: 5’- GTTTCGGGTCTGGATGCCTTCT-3’* |
