# Supplementary material for: Pretreatment with an antibiotics cocktail enhances the protective effect of probiotics by regulating SCFA metabolism and Th1/Th2/Th17 cell immune responses
Source: BMC Microbiol. 2024 Mar 18;24:91. doi: 10.1186/s12866-024-03251-2 (PMC10946100; doi:10.1186/s12866-024-03251-2)
Supplement: Supplementary file 5 — Supplementary Material 5 [file 12866_2024_3251_MOESM5_ESM.docx]

Table S2

The change of microbiome and SCFA levels in DSS, Abx+ DSS, Abx+ DSS+ CBM group

The relative abundance of the top 10 dominant bacteria taxa at the level of Phylum (Mean ± SD, %)

| Phylum | DSS | Abx+DSS | Abx+DSS+CBM |
| --- | --- | --- | --- |
| Bacteroidetes | 57.03±13.11 | 27.31±25.93 | 49.50±12.14 |
| Firmicutes | 31.75±11.73 | 27.80±13.89 | 36.15±19.52 |
| Proteobacteria | 3.84±2.75 | 28.75±22.15 | 1.32±1.67 |
| Verrucomicrobia | 0.20±0.51 | 19.97±7.96 | 12.04±7.77 |
| Actinobacteria | 0.28±0.57 | 1.06±1.70 | 0.45±0.49 |
| Epsilonbacteraeota | 5.55±5.17 | 4.33±1.08 | 0.02±0.01 |
| Planctomycetes | 0.09±0.26 | 1.06±2.03 | 0.14±0.20 |
| Acidobacteria | 0.04±0.07 | 0.24±0.26 | 0.10±0.19 |
| Chloroflexi | 0.05±0.15 | 0.24±0.54 | 0.02±0.03 |
| Gemmatimonadetes | 0.02±0.04 | 0.17±0.42 | 0.07±0.14 |

The relative abundance of the top 10 dominant bacteria taxa at the level of Order (Mean ± SD, %)

| Order | DSS | Abx+DSS | Abx+DSS+CBM |
| --- | --- | --- | --- |
| Bacteroidales | 56.97±13.16 | 27.01±26.19 | 49.48±12.15 |
| Clostridiales | 30.63±11.10 | 23.36±11.66 | 34.22±20.05 |
| Enterobacteriales | 0.77±1.34 | 23.25±20.52 | 0.92±1.56 |
| Verrucomicrobiales | 0.19±0.51 | 18.73±8.01 | 12.03±7.77 |
| Lactobacillales | 0.41±0.50 | 4.53±6.22 | 0.91±1.04 |
| Erysipelotrichales | 0.57±0.42 | 6.24±3.17 | 0.87±0.73 |
| Pseudomonadales | 0.14±0.39 | 3.09±1.85 | 0.03±0.03 |
| Campylobacterales | 5.55±5.17 | 1.20±1.08 | 0.02±0.01 |
| Pirellulales | 0.03±0.07 | 0.82±0.90 | 0.02±0.04 |
| Bacillales | 0.01±0.02 | 0.33±0.63 | 0.02±0.02 |

The relative abundance of the top 10 dominant bacteria taxa at the level of Family (Mean ± SD, %)

| Family | DSS | Abx+DSS | Abx+DSS+CBM |
| --- | --- | --- | --- |
| Bacteroidaceae | 29.82±13.59 | 18.59±18.18 | 16.76±9.58 |
| Muribaculaceae | 19.65±7.15 | 12.66±7.59 | 26.54±6.01 |
| Enterobacteriaceae | 0.77±1.34 | 16.75±20.52 | 0.92±1.56 |
| Lachnospiraceae | 15.94±5.37 | 14.34±1.51 | 22.97±14.70 |
| Akkermansiaceae | 0.18±0.51 | 10.66±8.05 | 12.02±7.77 |
| Ruminococcaceae | 13.02±7.94 | 5.35±3.93 | 9.95±5.66 |
| Clostridiaceae_1 | 0.01±0.00 | 8.32±9.38 | 0.08±0.04 |
| Prevotellaceae | 1.57±1.02 | 1.40±2.83 | 4.20±1.26 |
| Erysipelotrichaceae | 0.57±0.42 | 2.46±3.17 | 0.87±0.73 |
| Lactobacillaceae | 0.32±0.36 | 2.76±4.89 | 0.85±1.01 |

The relative abundance of the top 10 dominant bacteria taxa at the level of Genus (Mean ± SD, %)

| Genus | DSS | Abx+DSS | Abx+DSS+CBM |
| --- | --- | --- | --- |
| Bacteroides | 29.82±13.59 | 18.59±18.18 | 16.76±9.58 |
| Akkermansia | 0.18±0.51 | 14.62±8.05 | 12.02±7.77 |
| Citrobacter | 0.06±0.05 | 13.54±14.88 | 0.23±0.42 |
| Lachnospiraceae_NK4A136_group | 5.24±2.59 | 8.39±0.50 | 10.61±8.06 |
| Clostridium_sensu_stricto_1 | 0.01±0.00 | 9.35±9.91 | 0.07±0.04 |
| Escherichia-Shigella | 0.67±1.31 | 4.77±3.16 | 0.30±0.32 |
| Lactobacillus | 0.30±0.32 | 5.57±4.89 | 0.85±1.01 |
| Intestinimonas | 0.82±0.59 | 0.60±0.74 | 1.85±1.79 |
| Flavonifractor | 0.07±0.24 | 1.42±3.15 | 0.19±0.34 |
| Erysipelatoclostridium | 0.03±0.12 | 2.23±3.06 | 0.10±0.07 |

Fecal SCFA concentration among groups (Mean ± SD, ng/mg)

|  | DSS | Abx+DSS | Abx+DSS+CBM |
| --- | --- | --- | --- |
| Acetic.acid | 666.75±360.03 | 709.61±283.60 | 1137.67±381.77 |
| Propionic.acid | 264.58±135.16 | 186.50±174.72 | 429.21±135.74 |
| Isobutyric.acid | 15.67±5.60 | 18.32±2.61 | 13.69±4.80 |
| Butyric.acid | 279.31±235.35 | 196.89±267.55 | 382.20±213.69 |
| Isovaleric.acid | 14.71±6.72 | 14.41±4.70 | 10.32±3.93 |
| Valeric.acid | 28.75±11.14 | 6.44±14.15 | 13.10±16.04 |
| Caproic.acid | 1.10±0.22 | 1.72±1.40 | 1.42±0.56 |
| Total | 1270.87±698.48 | 1133.89±635.30 | 1987.59±712.44 |
