# Supplementary material for: Pretreatment with an antibiotics cocktail enhances the protective effect of probiotics by regulating SCFA metabolism and Th1/Th2/Th17 cell immune responses
Source: BMC Microbiol. 2024 Mar 18;24:91. doi: 10.1186/s12866-024-03251-2 (PMC10946100; doi:10.1186/s12866-024-03251-2)
Supplement: Supplementary file 6 — Supplementary Material 6 [file 12866_2024_3251_MOESM6_ESM.pdf]

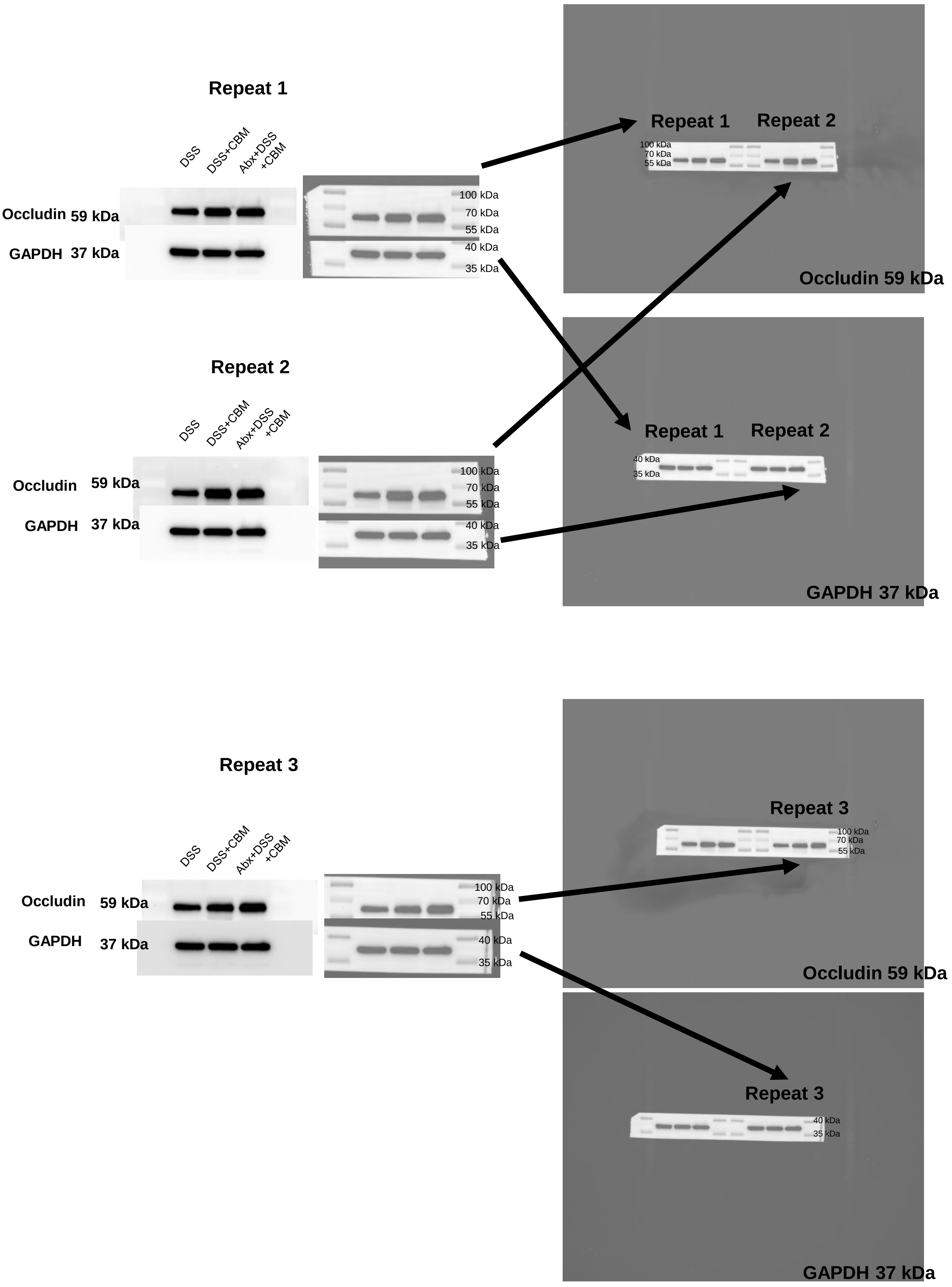

Figure S4. The original western blot images of Occludin.(10% gel, the band marker was #26616 from Thermos Fisher) These blot were cut prior to hybridization with antibodies during blotting. The target protein and the reference protein GAPDH which displayed were from the same pvdf membrane. Every specific antigen were duplicated for three times.

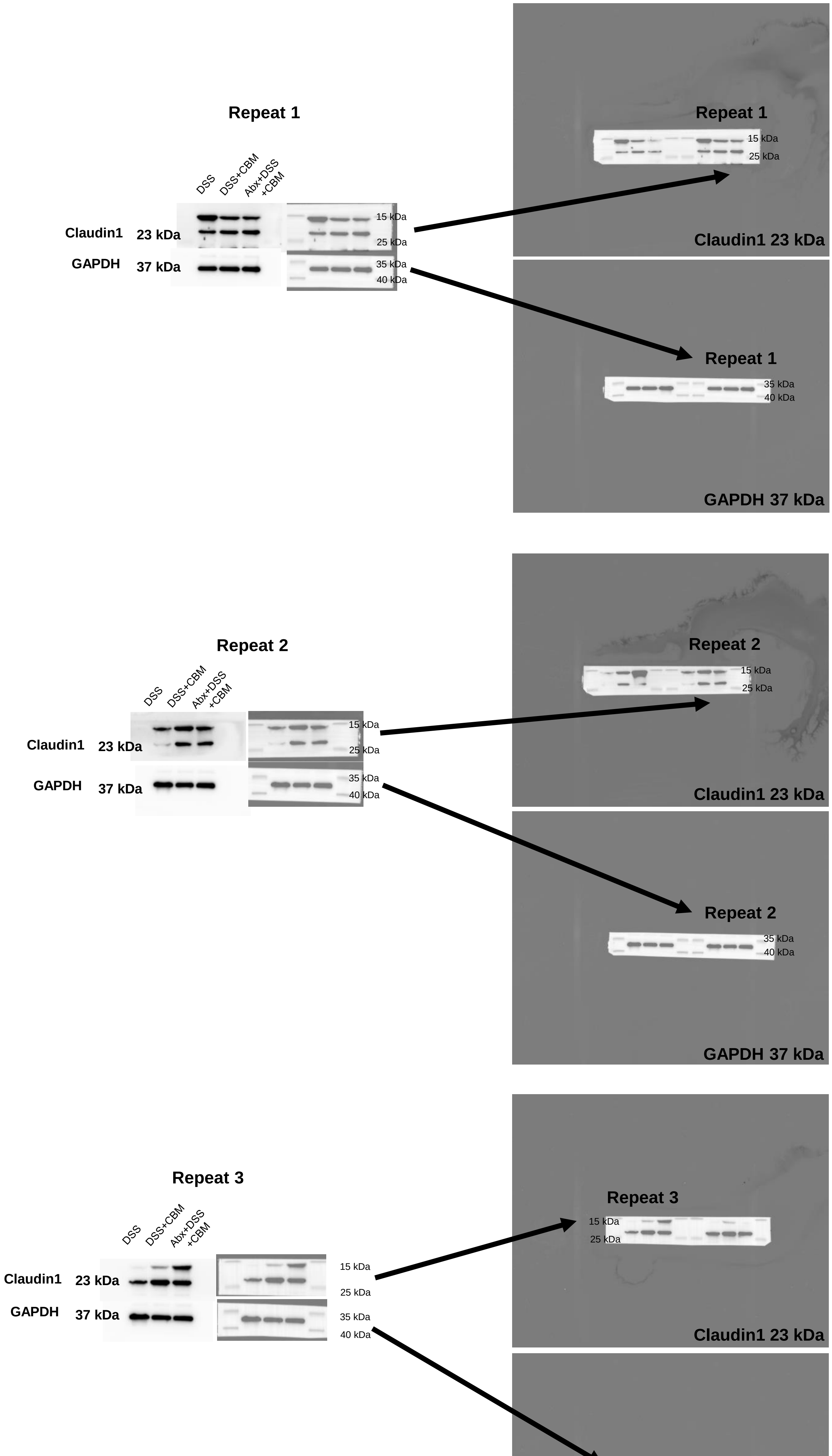

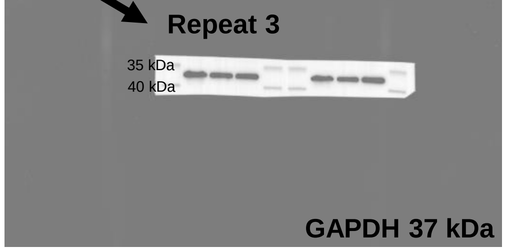

Figure S5. The original western blot images of Claudin1.(10% gel, the band marker was #26616 from Thermos Fisher) These blot were cut prior to hybridization with antibodies during blotting. The target protein and the reference protein GAPDH which displayed were from the same pvdf membrane. Every specific antigen were duplicated for three times.
